# Supplementary material for: Neutrophil breaching of the blood vessel pericyte layer during diapedesis requires mast cell-derived IL-17A
Source: Nat Commun. 2022 Nov 17;13:7029. doi: 10.1038/s41467-022-34695-7 (PMC9672103; doi:10.1038/s41467-022-34695-7)
Supplement: Supplementary file 1 — Supplementary Information [file 41467_2022_34695_MOESM1_ESM.pdf]

<u>Supplemental material</u>

Neutrophil breaching of the blood vessel pericyte layer during diapedesis requires mast cell-derived IL-17A

Joulia *et al.*

Corresponding author: Mathieu-Benoit Voisin, m.b.voisin@qmul.ac.uk

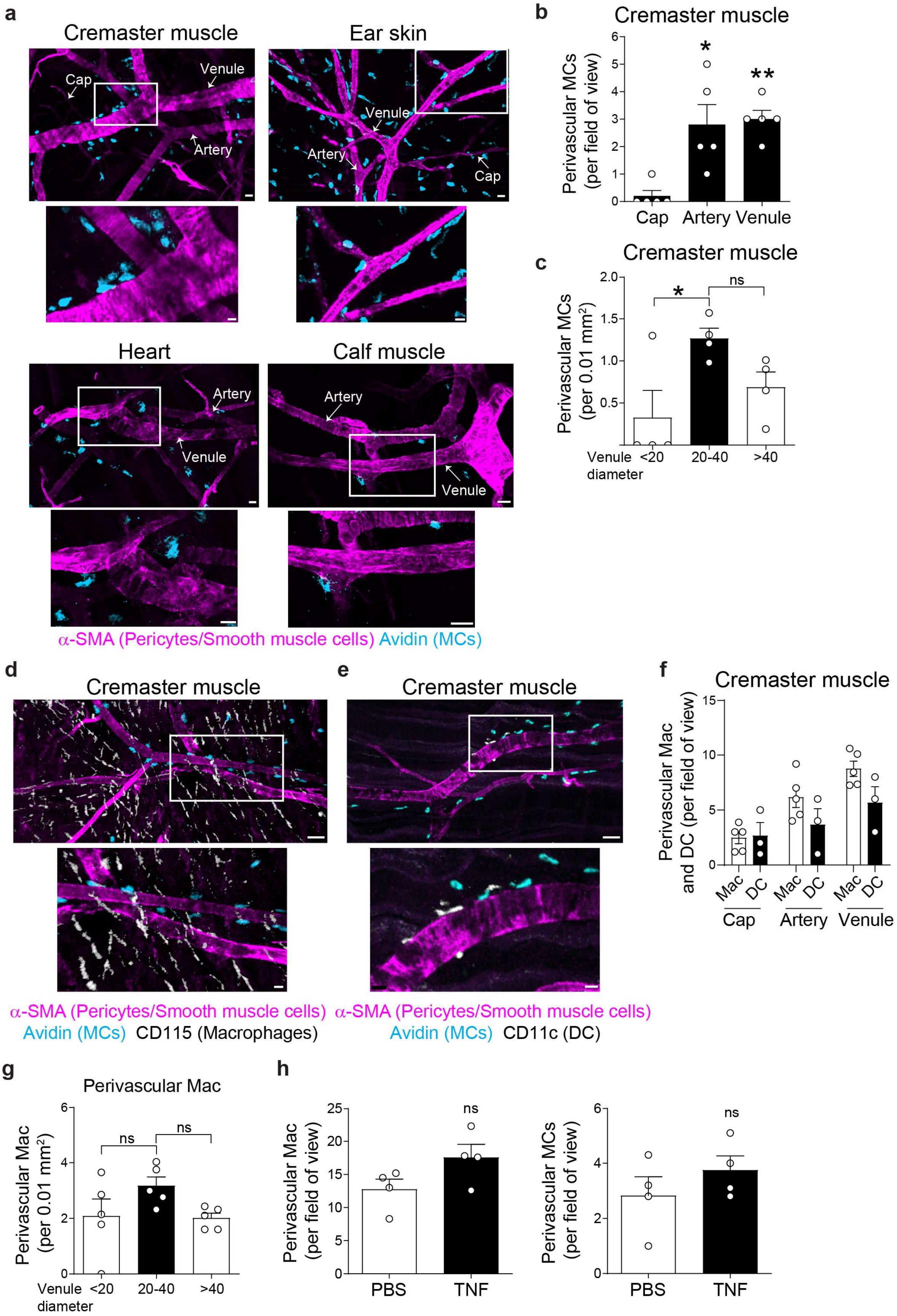
a
Cremaster muscle
Ear skin
Cap
Venule
Artery
Heart
Calf muscle
α-SMA (Pericytes/Smooth muscle cells) Avidin (MCs)
b
Cremaster muscle
Perivascular MCs (per field of view)
Cap
Artery
Venule
c
Cremaster muscle
Perivascular MCs (per 0.01 mm²)
ns
Venule diameter
<20
20-40
>40
d
Cremaster muscle
α-SMA (Pericytes/Smooth muscle cells)
Avidin (MCs) CD115 (Macrophages)
e
Cremaster muscle
α-SMA (Pericytes/Smooth muscle cells)
Avidin (MCs) CD11c (DC)
f
Cremaster muscle
Perivascular Mac and DC (per field of view)
Mac
DC
Cap
Artery
Venule
g
Perivascular Mac
Perivascular Mac (per 0.01 mm²)
ns
Venule diameter
<20
20-40
>40
h
Perivascular Mac (per field of view)
Perivascular MCs (per field of view)
ns
PBS
TNF

Supplementary Figure 1. Perivascular distribution of MCs, macrophages and DCs. a Representative confocal images of cremaster muscle, ear skin, heart and leg muscle sections immunostained with fluorescently labelled avidin and anti-α-SMA mAb to visualise mast cells and smooth muscle cells/pericytes, respectively, scale bars 20 μm (representative of 3 independent experiments). **b** Quantification of the number of perivascular MCs around capillary, artery and post-capillary venules (n= 5 mice, *p value=0.0397, **p value=0.0038). **c** Quantification of the number of perivascular MCs around different size of post-capillary venules (normalised to the venule diameter, n= 4 mice, *p value=0.0393). **d** and **e** Representative confocal images of whole mount cremaster muscles immunostained with fluorescently labelled avidin, anti-α-SMA and anti-CD115 or anti-CD11c mAbs and to visualise mast cells, smooth muscle cells/pericytes and macrophages (**d**) or DCs (**e**), respectively, scale bars 80 μm (representative of 3 independent experiments). **f** Quantification of the number of perivascular macrophages and DCs around capillary, artery and post-capillary venules (n= 5 mice for Mac and n=3 mice for DC). **g** Quantification of the number of perivascular macrophages around different size of post-capillary venules (normalised to the venule diameter, n= 5 mice). **h** Number of perivascular macrophages and MCs in PBS and TNF-stimulated WT mice (n= 4 mice). Mean±SEM (each mouse represents one independent experiment). **b, c** and **g** one-way ANOVA followed by Turkey's post-hoc test, **h** two-tailed Student's t-test; **b** and **h** Statistically significant differences from Cap or PBS or as indicated *p<0.05; **p<0.01. Source data are provided as a Source Data file.

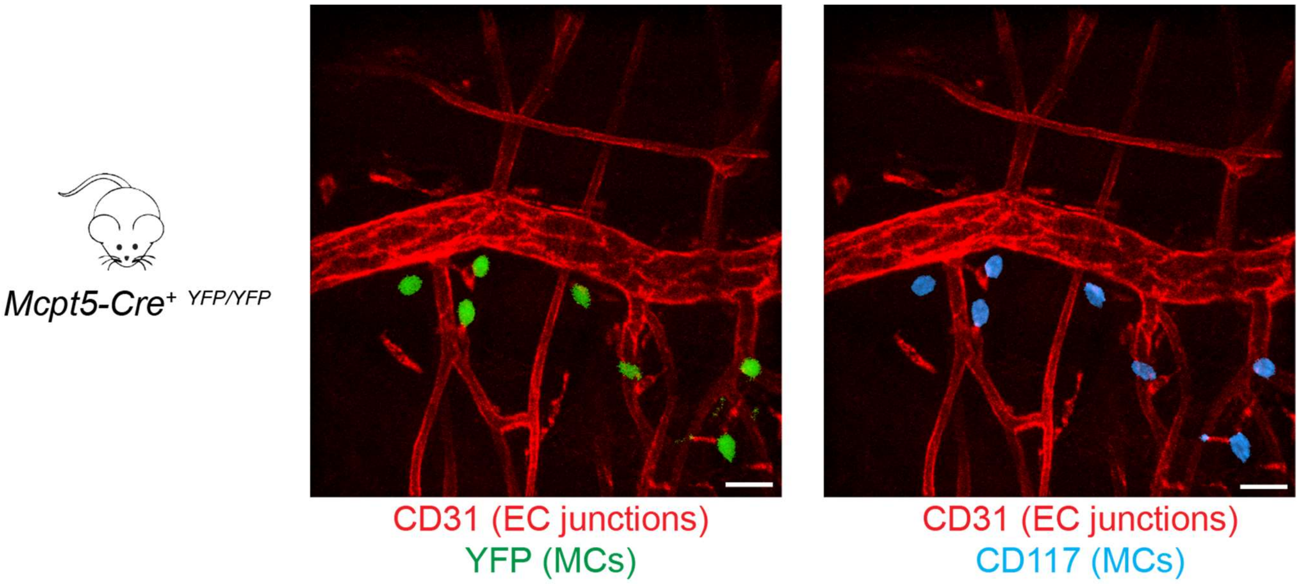

Supplementary Figure 2. Local injection of anti-CD117 labels all connective tissue MCs in the cremaster muscle. *Mcpt5-Cre*$^{YFP/YFP}$ mice were injected i.s. for 2h with AF555-labelled anti-CD31 mAb and AF647-labelled anti-CD117 mAb (10 µg) to visualise EC junctions (red) and MCs (blue), respectively. Representative confocal images of a cremasteric post-capillary venule at 2h post injection of the mAbs showing that 100% of YFP^{+} MCs are also stained with the anti-CD117 mAb, scale bars 30 µm (representative of 3 independent experiments). Source data are provided as a Source Data file.

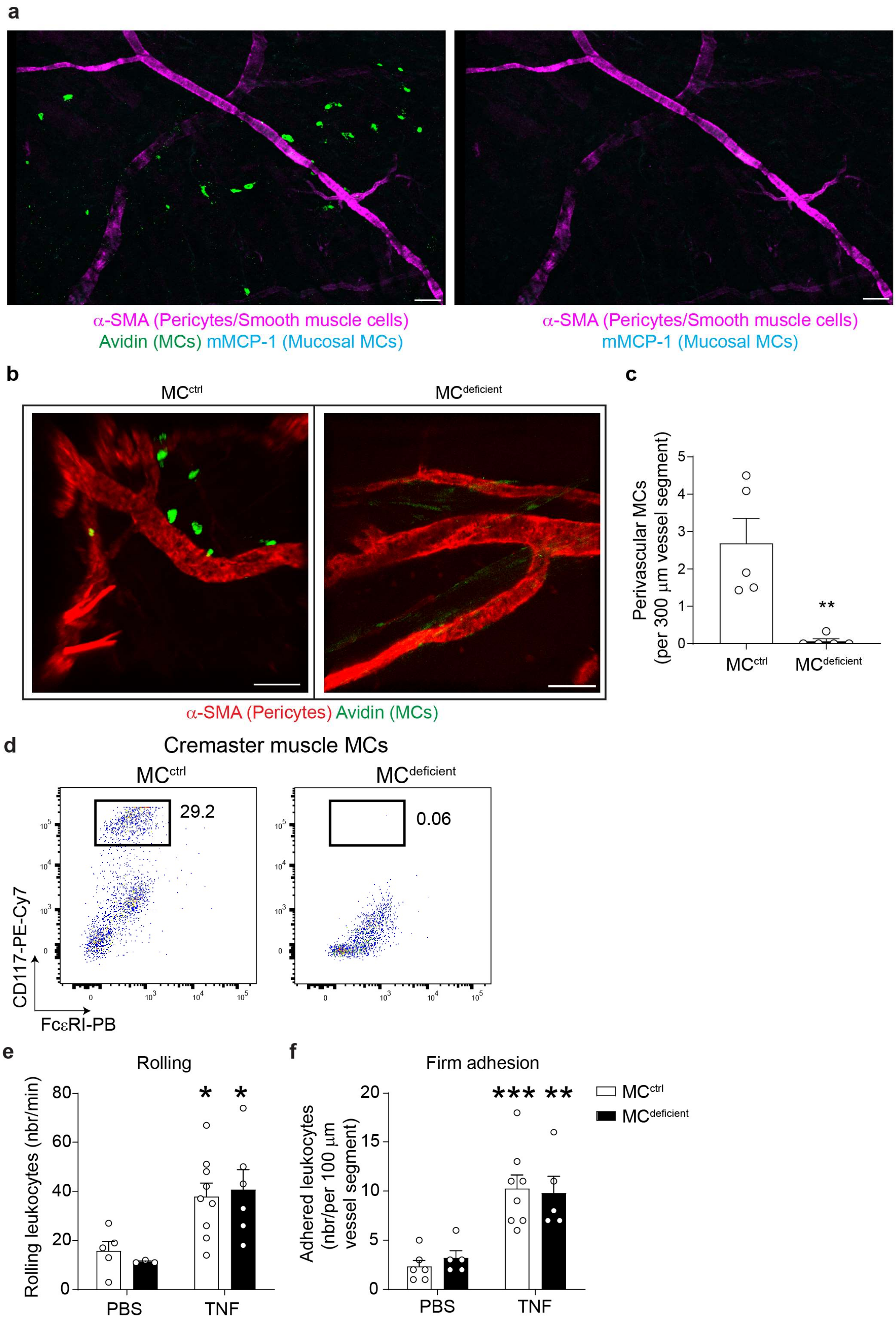

Supplementary Figure 3. MC deficient mice exhibit total absence of MCs and no defect in leukocyte rolling or adhesion. a Representative confocal image (n= 3 mice) of whole mount cremaster muscle immunostained with fluorescently labelled avidin, anti-α-SMA and anti-mMCP-1 (Mcpt-1) to visualise connective tissue MCs, smooth muscle cells/pericytes and mucosal MCs, respectively, showing the absence of mucosal MCs in the cremaster muscle, scale bars 100 µm. **b-c** Cremaster muscles from *Mcpt5-Cre*$^{-}$*-RDTA/RDTA* (MC^{ctrl}) or *Mcpt5-Cre*$^{+}$*-RDTA/RDTA* (MC^{deficient})

naive mice were collected and immunostained with fluorescently labelled avidin and anti-α-SMA mAb to visualise mast cells and pericytes, respectively. **b** Representative confocal images of whole mount cremaster muscle in MC^{ctrl} and $MC^{deficient}$ mice, scale bars 40 µm. **c** Quantification of the number of perivascular MCs around post-capillary venules in MC^{ctrl} and $MC^{deficient}$ mice (n= 5 mice, p value=0.0045). **d** Cremaster muscles of MC^{ctrl} and $MC^{deficient}$ mice were collected, dissociated and single cell suspensions were analysed by flow cytometry. Flow cytometry profiles show a complete absence of MCs ($CD117^+$,$FceRI^+$) in $MC^{deficient}$ mice. **e-f** *Mcpt5-Cre⁻-RDTA/RDTA* (MC^{ctrl}) or *Mcpt5-Cre⁺-RDTA/RDTA* ($MC^{deficient}$) mice were subjected to TNF-stimulation (300 ng; i.s. 2h) of the cremaster muscles. Tissues were surgically exteriorised and analysed for neutrophil-vessel wall interactions by brightfield IVM. **e** Quantification of the number of rolling leukocytes (cell number/min, n = 5 PBS MC^{ctrl} n=9 TNF MC^{ctrl} n=3 PBS $MC^{deficient}$ n= 6 $MC^{deficient}$, MC^{ctrl} *p value=0.0357, $MC^{deficient}$ *p value=0.0275). **f** Quantification of the number of adherent leukocytes per 100 µm length of post-capillary venules (n = 6 PBS MC^{ctrl} n=8 TNF MC^{ctrl} n=5 PBS $MC^{deficient}$ n= 5 $MC^{deficient}$, MC^{ctrl} ***p value=0.0002, $MC^{deficient}$ **p value=0.0059). Mean±SEM (each mouse represents one independent experiment). **c** two-tailed Student's t-test; **d,e** two-way ANOVA followed by Sidak's post-hoc test. Statistically significant differences from *Mcpt5-Cre⁻-RDTA* is indicated *p<0.05; **p<0.01; ***p<0.001. Source data are provided as a Source Data file.

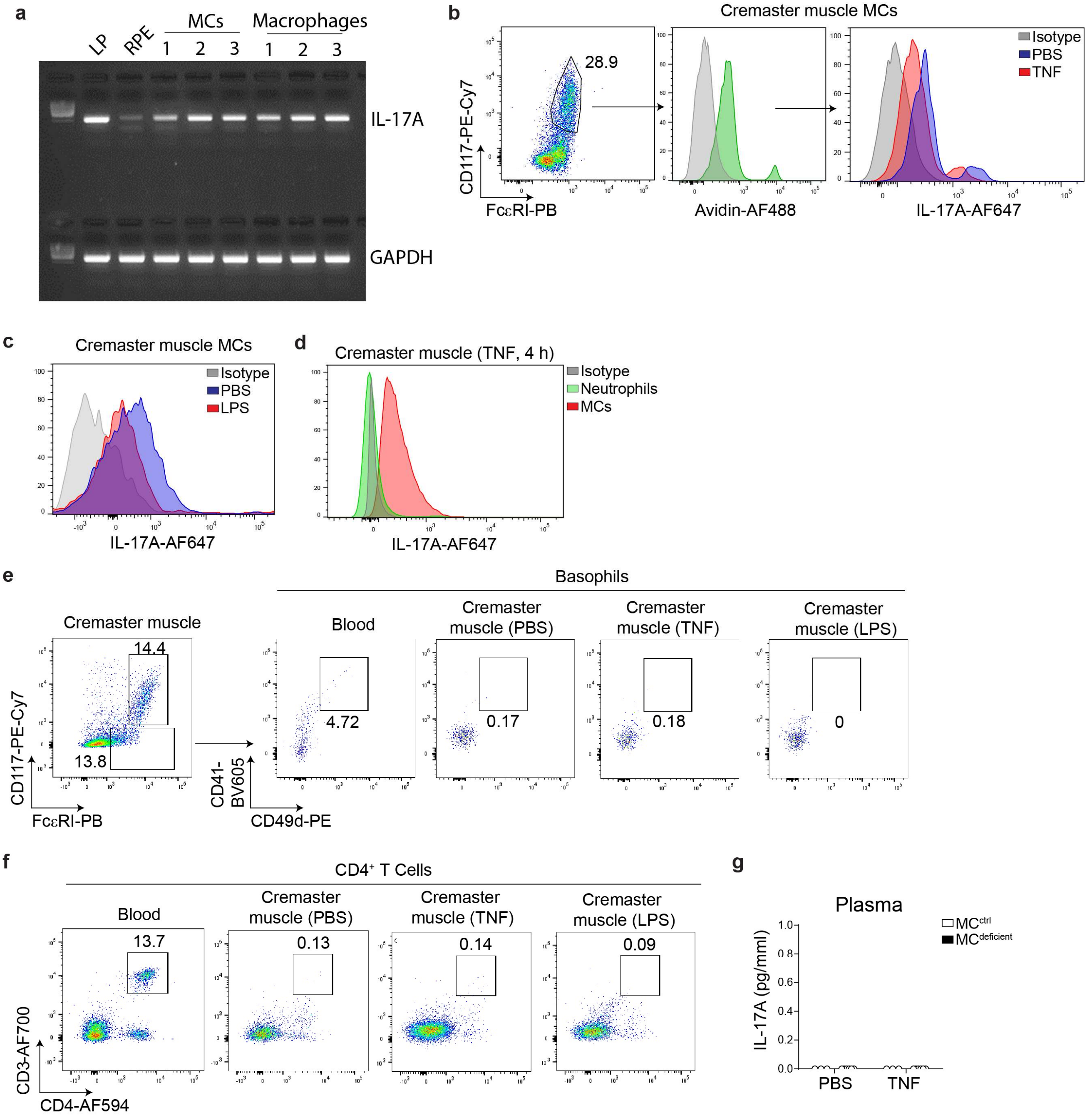

Supplementary Figure 4. Expression of IL-17A in MCs, neutrophils, basophils and CD4$^+$ T cells. **a** MCs from WT cremaster muscles were isolated by flow cytometry cell sorting (CD45$^+$, FcεRI$^+$, CD117$^+$), and the presence of IL-17A mRNA was analysed by RT-PCR. Gut lamina propria (LP) and retinal pigment epithelium were used as positive and negative controls, respectively (MC and macrophages flow cytometry sorted from n=3 independent mice). **b-f** Cremaster muscles of PBS, TNF or LPS-stimulated WT mice were collected, dissociated and single cell suspensions were analysed by flow cytometry. Histograms show IL-17A expression in MCs (**b-c**, CD45$^+$, FcεRI$^+$, CD117$^+$, avidin$^+$), neutrophils (**d**, CD45$^+$, LY6G$^+$). **e** and **f** Representative flow cytometry profiles showing the absence of recruitment of basophils (**e**) and CD4$^+$ T cells (**f**) in the cremaster muscles

following TNF or LPS stimulation. **g** Plasma level of IL-17A analysed by ELISA in PBS and TNF treated (4h) MC^{ctrl} or MC^{deficent} (n=3 PBS MC^{ctrl} mice, n=3 PBS MC^{deficient} mice, n=6 TNF MC^{ctrl} mice and n=6 TNF MC^{deficient} mice). Mean±SEM (each mouse represents one independent experiment). Number indicated percentage of positive cells amongst the parent gate. Source data are provided as a Source Data file.

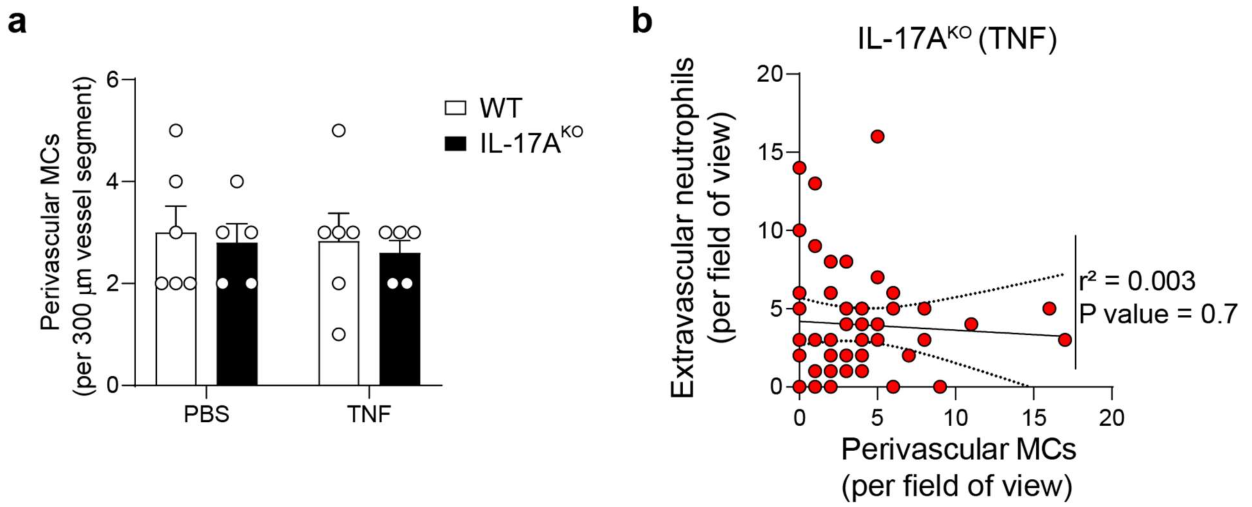

Supplementary Figure 5. IL-17A^{KO} and WT (control) mice exhibit similar levels of MCs in the cremaster muscles. a Number of perivascular MCs in PBS and TNF-stimulated WT and IL-17A^{KO} mice (n= 6 PBS WT n= 5 PBS IL-17A^{KO} n= 6 TNF WT n=5 IL-17A^{KO} mice) as quantified by confocal microscopy. **b** Correlation of the number of extravascular neutrophils and perivascular MCs in the cremaster muscle of TNF-stimulated IL-17A^{KO} mice (n= 49 venules, data pooled from 5 mice). Line indicated linear regression and dashed lines 95% confidence band (r^2=0.003). Mean±SEM (each mouse represents one independent experiment). **b** Spearman's rank correlation test. Source data are provided as a Source Data file.

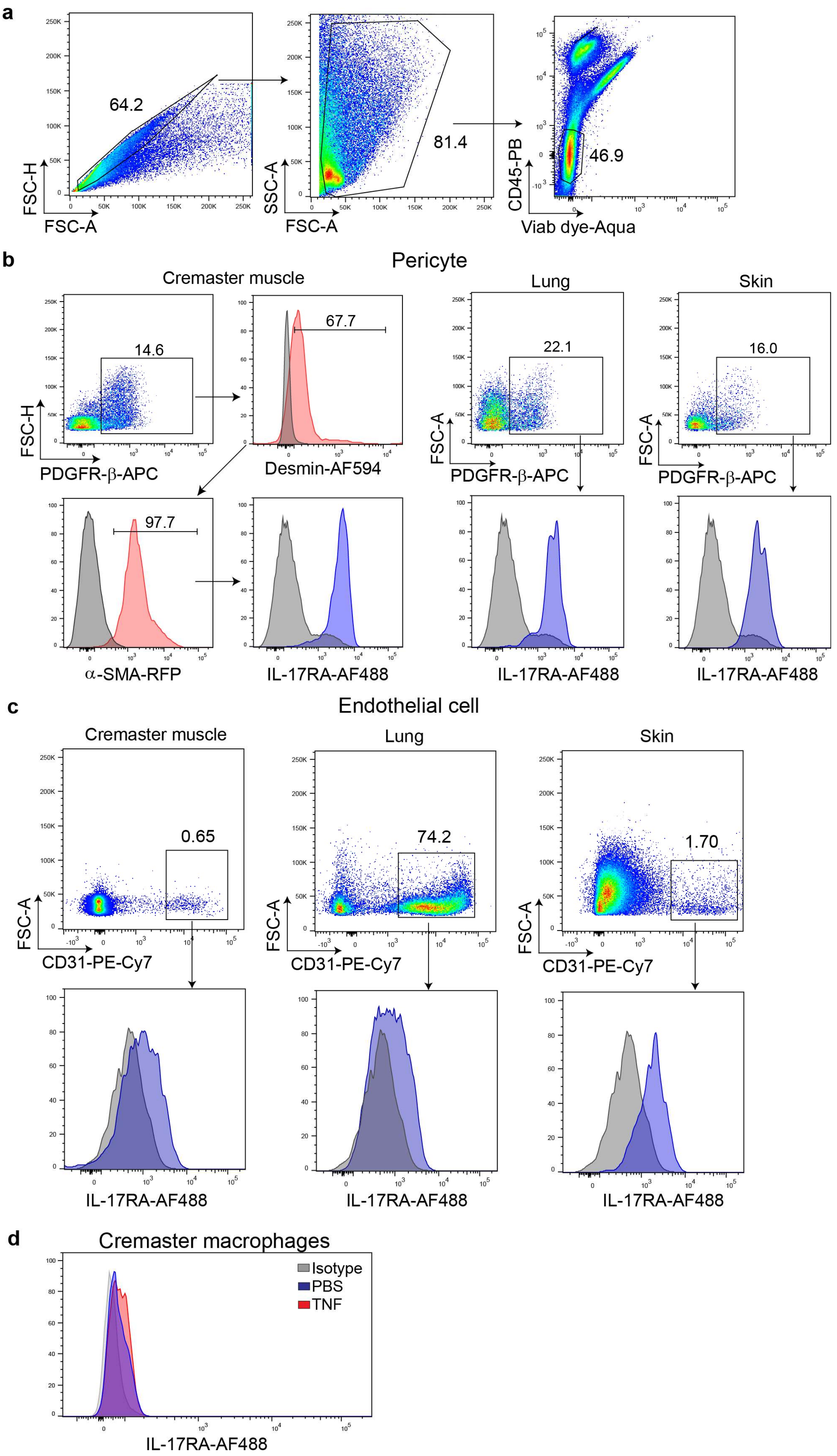
a
64.2
81.4
46.9
FSC-H
FSC-A
SSC-A
CD45-PB
Viab dye-Aqua
b
Pericyte
Cremaster muscle
Lung
Skin
14.6
67.7
22.1
16.0
97.7
FSC-H
FSC-A
PDGFR-β-APC
Desmin-AF594
α-SMA-RFP
IL-17RA-AF488
c
Endothelial cell
Cremaster muscle
Lung
Skin
0.65
74.2
1.70
FSC-A
CD31-PE-Cy7
IL-17RA-AF488
d
Cremaster macrophages
Isotype
PBS
TNF
IL-17RA-AF488

Supplementary Figure 6. Flow cytometry gating strategy for ECs and pericytes and IL-17RA expression levels. a-c Cremaster muscles, ear skin and lungs of α-SMA-RFP$^+$ mice were collected, dissociated and analysed by flow cytometry. **a** Representative flow cytometry profiles showing the gating strategy to exclude doublets, CD45$^+$ and dead cells. **b** Gating strategy and profile of IL-17RA expression by pericytes from cremaster muscles, lungs and ear skin. **c** Gating strategy and profile of IL-17RA expression by ECs from cremaster muscles, lungs and ear skin. Numbers indicated the percentage of cells within the gate of interest. **d** Profile of IL-17RA expression by macrophages (CD45$^+$ CD115$^+$) from cremaster muscles in PBS or TNF stimulated mice. Representative of n= 6 mice (each mouse represents one independent experiment). Source data are provided as a Source Data file.

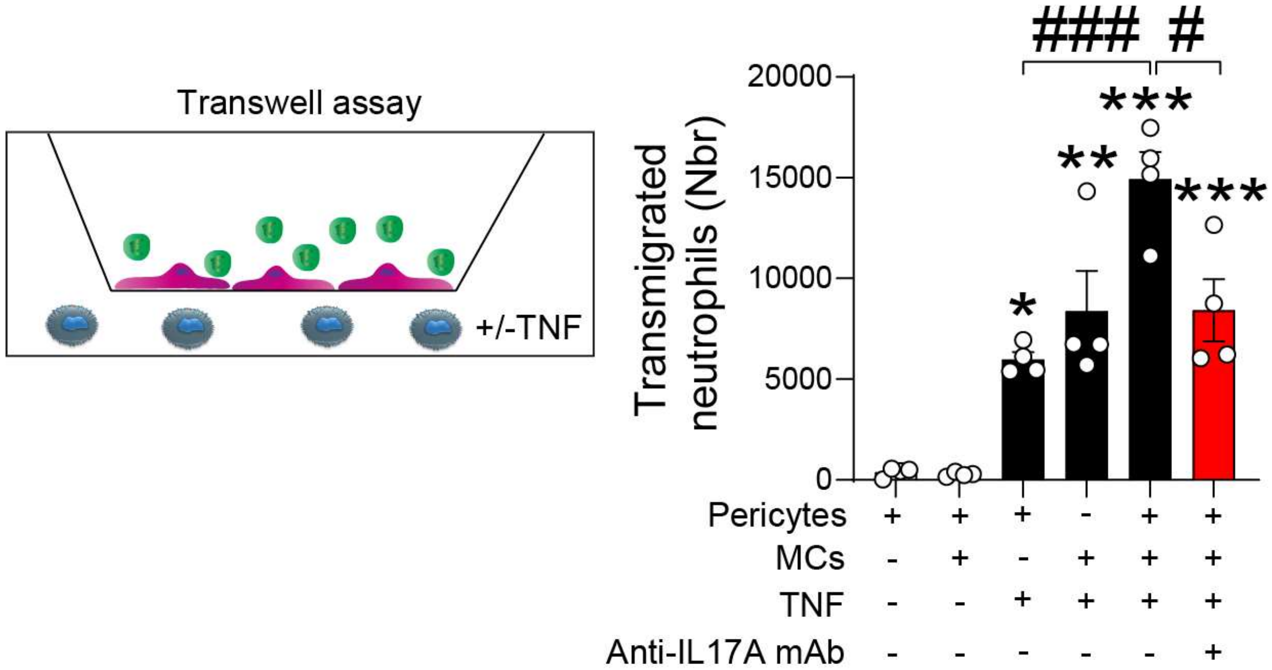

Supplementary Figure 7. Mast cells induce neutrophil migration through pericyte in vitro. Isolated primary cremaster muscle pericytes and MCs were grown on the top or bottom chamber chambers respectively. TNF or PBS (+/- anti-IL17A mAb) was added to the bottom chamber and isolated blood neutrophils were placed in the top chamber for 1 h. Number of neutrophils in the bottom was determined by flow cytometry. Mean±SEM. one-way ANOVA followed by Tukey's post-hoc test Statistically significant differences from PBS are indicated by *p<0.05, **p<0.01, ***p<0.001 or as indicated by #p<0.05, ###p<0.001. Source data are provided as a Source Data file.

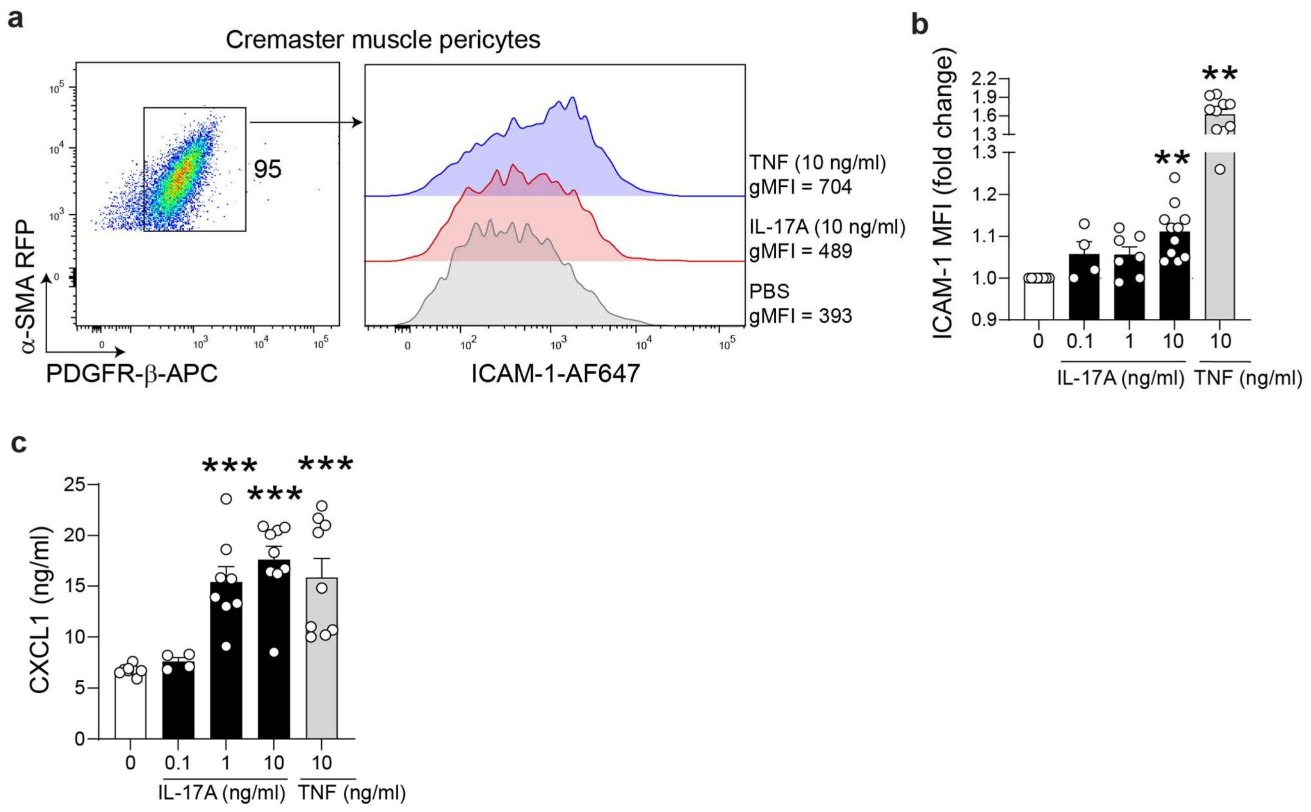

Supplementary Figure 8. IL17A directly regulates pericyte ICAM-1 and CXCL1 expression in vitro. Isolated primary cremaster pericytes from α-SMA-RFP^{+} mice were stimulated for 6 h with IL-17A or TNF. Surface ICAM-1 expression CXCL1 release in supernatant were analysed by flow cytometry and ELISA, respectively. **a** Representative flow cytometry profiles of isolated pericytes (PDGFR-β^{+} α-SMA-RFP^{+}) their surface expression of ICAM-1 (histogram, right panel) following indicated stimulation. Geometric mean of fluorescence intensity (gMFI) for each stimulating condition is indicated next to the histogram plot. The number in the dot plot shows the purity of pericyte isolation. **b** Quantification of ICAM-1 gMFI represented as fold change to PBS condition (n= 8 0 ng/ml IL-17A n= 4 0.1 ng/ml IL-17A n= 7 1 ng/ml IL-17A n= 11 10 ng/ml IL-17A n=10 10 ng/ml TNF; data pooled from 5 independent experiments, 10 ng/ml IL-17A **p value=0.0036, 10 ng/ml TNF **p value=0.0017). **c** CXCL1 level in supernatants quantified by ELISA ((n= 8 0 ng/ml IL-17A n= 4 0.1 ng/ml IL-17A n= 8 1 ng/ml IL-17A n= 9 10 ng/ml IL-17A n=9 10 ng/ml TNF; data pooled from 5 independent experiments, 1 ng/ml IL-17A ***p value=0.0009, 10 ng/ml IL-17A ***p value<0.0001, 10 ng/ml TNF ***p value=0.0003). Mean±SEM. **b,c** one-way ANOVA followed by Tukey's post-hoc test Statistically significant differences from PBS are indicated by **p<0.01, ***p<0.001. Source data are provided as a Source Data file.

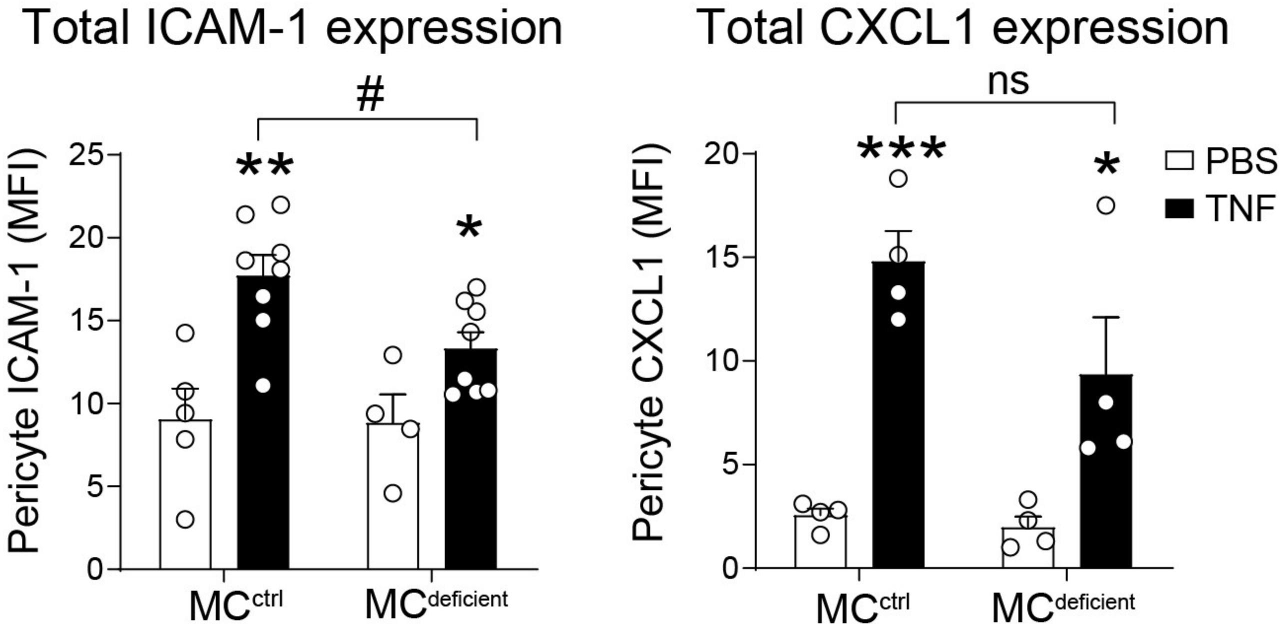

Supplementary Figure 9. Global pericyte ICAM-1 and CXCL1 expression is not impacted by the absence of MCs. Cremaster muscles of MC^{ctrl} or MC^{deficent} mice were stimulated with TNF (300 ng) or PBS for 4h. Cremaster muscles were collected and immunostained for MCs (avidin), pericytes (α-SMA) and ICAM-1 or CXCL1. Quantification of ICAM-1 MFI (n=5 PBS MC^{ctrl} mice, n=8 TNF MC^{ctrl} mice, n=4 PBS MC^{deficient} mice and n=8 TNF MC^{deficient} mice, **p value=0.004, *p value=0.0431, #p value=0.0341) and CXCL1 MFI (n=4 mice per group, ***p value=0.0003, *p value=0.0134) on pericytes. Mean±SEM (each mouse represents one independent experiment). two-way ANOVA followed by Sidak's post-hoc test. *p<0.05, **p<0.01, ***p<0.001 as compared to MC^{ctrl} or as indicated by #p<0.05 (ns= not significant). **Source data are provided as a Source Data file.**

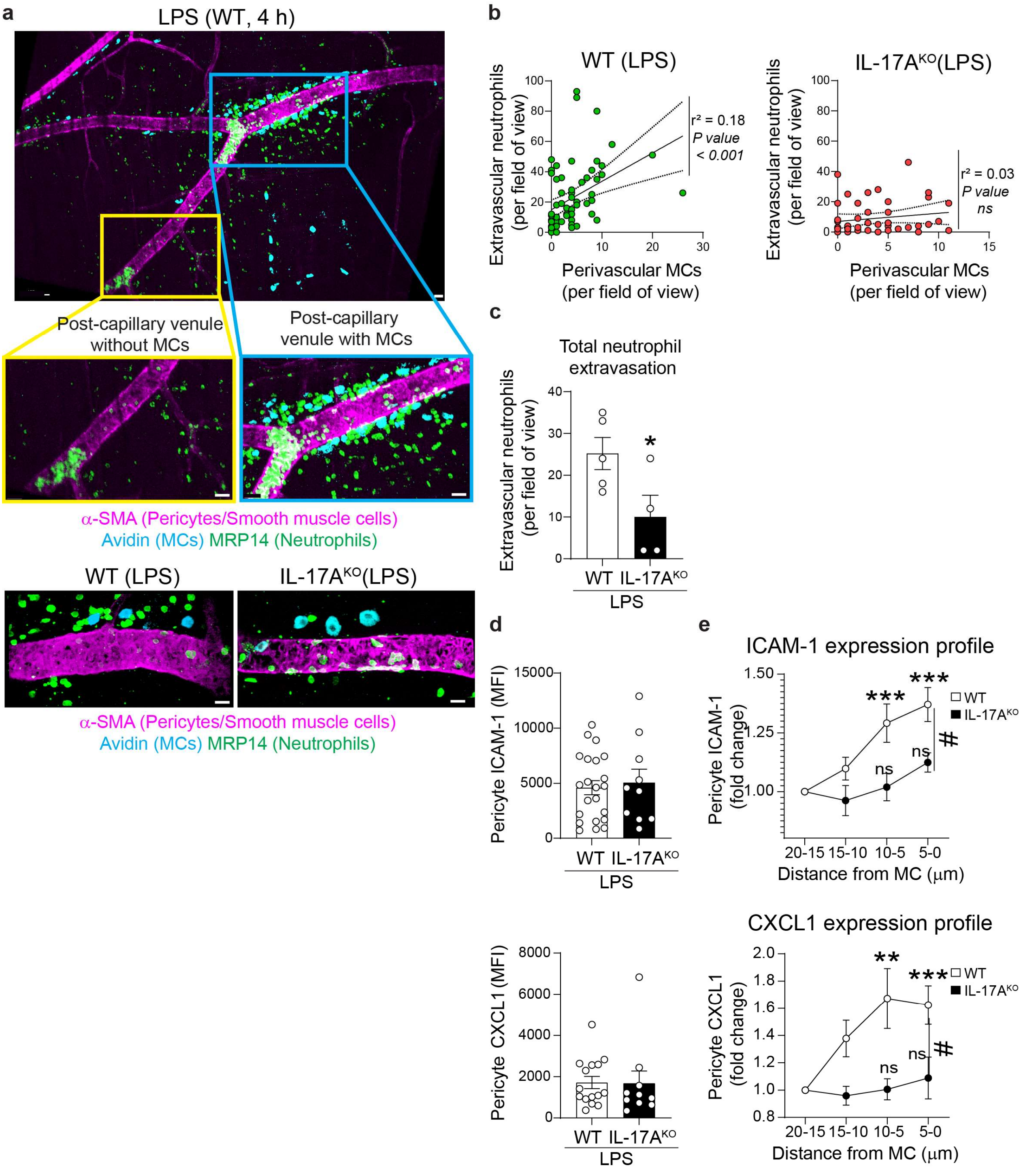

Supplementary Figure 10. Perivascular MCs promote neutrophil migration and graded ICAM-1 and CXCL1 expression following LPS stimulation. Cremaster muscles of WT or IL-17A^{KO} mice were stimulated with LPS (300 ng) for 4h. Tissues were immunostained for neutrophils (MRP14), MCs (avidin), pericytes/smooth muscle cells (α-SMA) and ICAM-1 or CXCL1. **a** Mouse cremaster 4h post LPS-stimulation in WT or IL-17A^{KO} mice, image representative of n=5 mice, scale bars 20 µm. **b** Correlation between the number of extravascular neutrophils and perivascular MCs (WT, n=64

venules) or ear skin (IL-17A^{KO}, n=45 venules); data pooled from 4 mice. Lines indicated linear regression and dashed lines 95% confidence band. **c** Total neutrophil extravasation (n=5 WT and n=4 IL-17A^{KO} mice, p value=0.0473). **d** Quantification of ICAM-1 MFI (n=22 WT, n=10 IL-17A^{KO} vessel segments) and CXCL1 MFI (n=15 WT, n=10 IL-17A^{KO} vessel segments) on pericytes. **e** Quantification of pericyte ICAM-1 MFI in 5 µm-wide consecutive regions from a perivascular MC in LPS treated WT or IL-17A^{KO} mice (n=30 WT and n=14 IL-17A^{KO}, perivascular MC regions, data pooled from 3 mice, 10-5 ***p value=0.0007, 5-0 ***p value<0.0001, 5-0 #p value=0.0285) pericyte CXCL1 MFI (n=23 WT and n=20 IL-17A^{KO}, perivascular MC regions, data pooled from 3 mice, 10-5 **p value=0.0022, 5-0 ***p value=0.0009, 5-0 #p value=0.01). Mean±SEM (each mouse represents one independent experiment). **b** Spearman's rank correlation test; **c** two-tailed Student's t-test; **d** two-way ANOVA followed by Sidak's post-hoc test. *p<0.05, **p<0.01, ***p<0.001 as compared to 20-15 region or as indicated by #p<0.05 (ns= not significant). Source data are provided as a Source Data file.

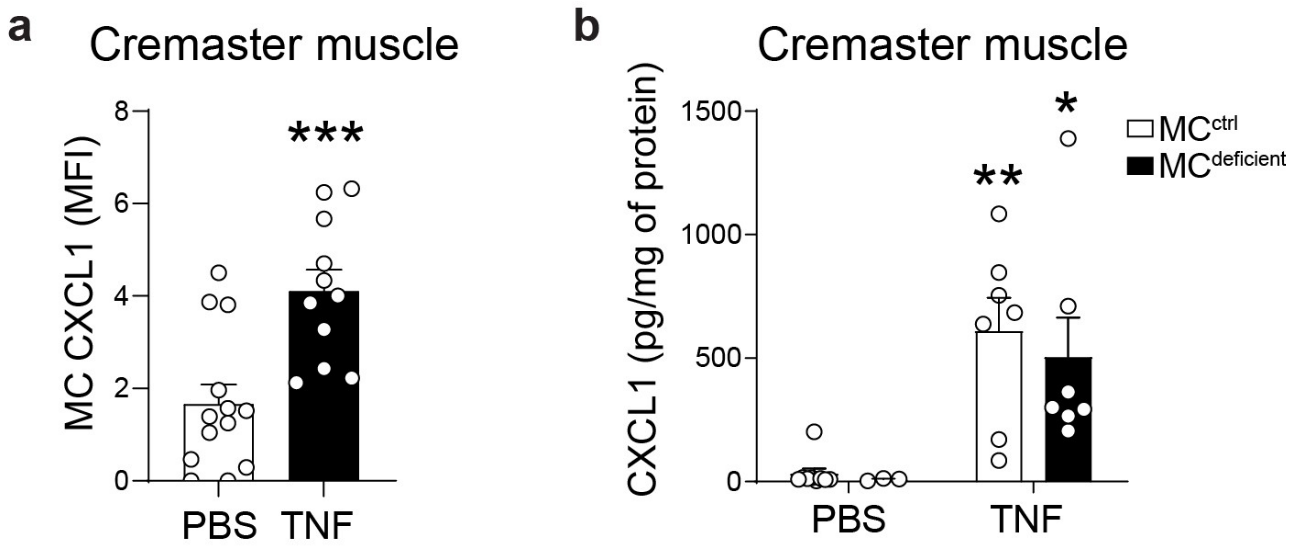

Supplementary Figure 11. TNF induces CXCL1 expression by MCs but CXCL1 derived from MCs is not essential for overall tissue production. Cremaster muscles of WT mice were stimulated with TNF (300 ng) or PBS for 4h. Cremaster muscles were collected and immunostained for MCs (avidin), pericytes (α-SMA) or CXCL1. **a** Quantification of CXCL1 MFI in perivascular MCs (n=13 PBS and n=11 TNF mice, p value=0.0007). **b** Tissue level of CXCL1 analysed by ELISA in PBS and TNF treated (4h) MC^{ctrl} or $MC^{deficent}$ (n=9 PBS MC^{ctrl} mice, n=3 PBS $MC^{deficient}$ mice, n=7 TNF MC^{ctrl} mice and n=7 TNF $MC^{deficient}$ mice, **p value=0.0015, *p value=0.0229). Mean±SEM (each mouse represents one independent experiment). **a** two-tailed Student's t-test, **b** two-way ANOVA followed by Sidak's post-hoc test. *p<0.05, **p<0.01 as compared to PBS. Source data are provided as a Source Data file.
