# Supplementary material for: Neutrophil breaching of the blood vessel pericyte layer during diapedesis requires mast cell-derived IL-17A
Source: Nat Commun. 2022 Nov 17;13:7029. doi: 10.1038/s41467-022-34695-7 (PMC9672103; doi:10.1038/s41467-022-34695-7)
Supplement: Supplementary file 3 — Description of Additional Supplementary Files [file 41467_2022_34695_MOESM3_ESM.pdf]

Description of Additional Supplementary Files

File Name: Supplementary Movie 1
Description: **Related to Fig. 1. Neutrophil TEM is independent of perivascular MC localisation.** The video captures a post-capillary venule 2 h following TNF stimulation (300 ng) in a *LysM-EGFP-ki; α-SMA-RFPcherry-Tg* mouse that displays GFP^{high} neutrophils and RFP^{+} smooth muscle cell and pericytes. EC junctions and MCs were labelled in vivo with an AF532-anti-CD31 mAb (dark blue) and an AF647-CD117 mAb (light blue), respectively. The video shows luminal and interstitial views of GFP^{bright} neutrophils (green) interacting with ECs and pericytes (magenta). The movie illustrates the sites of TEM and their absence of association with the localisation of perivascular MCs.

File Name: Supplementary Movie 2
Description: **Related to Fig. 1. Neutrophil subendothelial migration directed towards Perivascular MCs.** The video captures a post-capillary venule 2 h following TNF stimulation (300 ng) in a *LysM-EGFP-ki; α-SMA-RFPcherry-Tg* mouse that displays GFP^{high} neutrophils and RFP^{+} smooth muscle cell and pericytes. EC junctions and MCs were labelled in vivo with an AF532-anti-CD31 mAb (dark blue) and an AF647-CD117 mAb (light blue), respectively. The video shows luminal and interstitial views of GFP^{bright} neutrophils (green) interacting with ECs and pericytes (magenta). The movie illustrates the location of trans-pericyte migration sites (TPMs) along the vessel segment.

File Name: Supplementary Movie 3
Description: **Related to Fig. 1g. Neutrophil TEM and sub-EC migration in a TNF-stimulated venule in close apposition to MCs.** The video captures a post-capillary venule 2 h following TNF stimulation (300 ng) in a *LysM-EGFP-ki; α-SMA-RFPcherry-Tg* mouse that displays GFP^{high} neutrophils and RFP^{+} smooth muscle cell and pericytes. EC junctions and MCs were labelled in vivo with an AF532-anti-CD31 mAb (dark blue) and an AF647-CD117 mAb (light blue), respectively. The video shows luminal and interstitial views of GFP^{bright} neutrophil (green) interacting with ECs and pericytes (magenta). The movie illustrates the migratory path of a neutrophil following paracellular TEM and subsequent efficient migration in the sub-EC space directed towards a perivascular MC. The neutrophil was isolated from the inflammatory response for improved clarity by creating an isosurface using Imaris software. Still images of this video are shown in Figure 1g.

File Name: Supplementary Movie 4
Description: **Related to Fig. 1i. Neutrophil TPM migration in a TNF-stimulated venule in close apposition to MCs.** The video captures a post-capillary venule 2 h following TNF stimulation (300 ng) in a *LysM-EGFP-ki; α-SMA-RFPcherry-Tg* mouse that displays GFP^{high} neutrophils and RFP^{+} smooth muscle cell and pericytes. EC junctions and MCs were labelled in vivo with an AF532-anti-CD31 mAb (dark blue) and an AF647-CD117 mAb (light blue), respectively. The video shows GFP^{bright} neutrophils (green) exiting the blood vessel wall through the pericytes layer (magenta) as visualised from the interstitial side. The movie illustrates the migratory paths and directionality (arrows) of extravasating neutrophils from their site of TPM migration to their motility within the interstitium; and shows the preponderance of neutrophil TPM sites in the vicinity of perivascular MCs. Neutrophil paths and directionality were generated manually using the spot function of Imaris software.

File Name: Supplementary Movie 5
Description: **Related to Fig. 1i. Neutrophil TPM hotspot in the vicinity of a perivascular MC.** The confocal IVM video shows a cremaster muscle post-capillary venule of a *LysM-EGFP-ki; α-SMA-RFPcherry-Tg* mouse in which the TPM of GFP^{bright} neutrophils (green) are tracked following TNF stimulation (300 ng, 2h). EC junctions and MCs were labelled in vivo with an AF532-anti-CD31 mAb (dark blue) and an AF647-CD117 mAb (light blue), respectively. The video shows the interstitial view of selected neutrophils undergoing TPM migration near a perivascular MC and their physical interaction with the latter. Lines indicated migratory paths of neutrophils. Neutrophils were isolated from the inflammatory response for improved clarity by creating an isosurface using Imaris software. Still images of this video are shown in Figure 1i.

File Name: Supplementary Movie 6
Description: **Related to Fig. 2. Neutrophil TEM and TPM migration in a TNF-stimulated venule in $MC^{deficient}$ mice.** The video captures a post-capillary venule following TNF stimulation (300 ng) in a *Mcpt5-Cre^{+}-RDTA;LysM-EGFP-ki; α-SMA-RFPcherry-Tg* mouse ($MC^{deficient}$) that displays GFP^{high} neutrophils, RFP^{+} smooth muscle cell and pericytes and no MCs. EC junctions and MCs were labelled in vivo with an AF532-anti-CD31 mAb (dark blue) and an AF647-CD117 mAb (light blue), respectively. The video shows luminal and interstitial views of a GFP^{bright} neutrophil (green) interacting with ECs (dark blue) and pericytes (magenta). The movie illustrates that whilst the number of neutrophil TEM is normal in $MC^{deficient}$, the number of TPM is greatly reduced. Surfaces of neutrophil TEM & TPM events were generated manually using the isosurface function of Imaris software and are shown independently from the rest of the non-extravasating neutrophils for clarity.

File Name: Supplementary Movie 7
Description: **Related to Fig. 2c. Neutrophil sub-EC migration defect in $MC^{deficient}$ mice.** The video captures a post-capillary venule following TNF stimulation (300 ng) in a *Mcpt5-Cre^{+}-RDTA;LysM-EGFP-ki; α-SMA-RFPcherry-Tg* mouse ($MC^{deficient}$) that displays GFP^{high} neutrophils, RFP^{+} smooth muscle cell and pericytes and no MCs. EC junctions and MCs were labelled in vivo with an AF532-anti-CD31 mAb (dark blue) and an AF647-CD117 mAb (light blue), respectively. The video shows luminal and interstitial views of a GFP^{bright} neutrophil (green) interacting with ECs (dark blue) and pericytes (magenta). The movie illustrates that neutrophil TEM occurs normally independently of MCs. However once in the sub-EC space, the neutrophil loses directionality. Finally, the neutrophil undergoes TPM but remains stationary on the pericyte surface. The neutrophil was isolated from the inflammatory response for improved clarity by creating an isosurface using Imaris software. Still images of this video are shown in Figure 2c.

File Name: Supplementary Movie 8
Description: **Related to Fig. 3. Neutrophil TEM and TPM migration in a TNF-stimulated venule following anti-IL-17A mAb treatment.** The video captures a post-capillary venule 2 h following TNF stimulation (300 ng) and anti-IL-17A mAb in a *LysM-EGFP-ki; α-SMA-RFPcherry-Tg* mouse that displays GFP^{high} neutrophils and RFP^{+} smooth muscle cell and pericytes.. EC junctions and MCs were labelled in vivo with an AF532-anti-CD31 mAb (dark blue) and an AF647-CD117 mAb (light blue), respectively. The video shows luminal and interstitial views of a GFP^{bright} neutrophil (green) interacting with ECs (dark blue) and

pericytes (magenta). The movie illustrates that whilst the number of neutrophil TEM is normal, the number of TPM is reduced following anti-IL-17A mAb treatment. Surfaces of neutrophil TEM & TPM events were generated manually using the isosurface function of Imaris software and are shown independently from the rest of the non-extravasating neutrophils for clarity.

File Name: Supplementary Movie 9
Description: **Related to Fig. 3. Neutrophil sub-EC migration following anti-IL-17A mAb treatment.** The video captures a post-capillary venule 2 h following TNF stimulation (300 ng) and anti-IL-17A mAb in a *LysM-EGFP-ki; α-SMA-RFPcherry-Tg* mouse that displays GFP^{high} neutrophils and RFP^{+} smooth muscle cell and pericytes. EC junctions and MCs were labelled in vivo with an AF532-anti-CD31 mAb (dark blue) and an AF647-CD117 mAb (light blue), respectively. The video shows luminal and interstitial views of a GFP^{bright} neutrophil (green) interacting with ECs (dark blue) and pericytes (magenta). The movie illustrates that following TEM and once in the sub-EC space, the neutrophil loses directionality and does not undergo TPM. Surfaces of neutrophil TEM & TPM events were generated manually using the isosurface function of Imaris software and are shown independently from the rest of the non-extravasating neutrophils for clarity.
