# Supplementary material for: Neutrophil breaching of the blood vessel pericyte layer during diapedesis requires mast cell-derived IL-17A
Source: Nat Commun. 2022 Nov 17;13:7029. doi: 10.1038/s41467-022-34695-7 (PMC9672103; doi:10.1038/s41467-022-34695-7)
Supplement: Supplementary file 13 — Reporting Summary [file 41467_2022_34695_MOESM13_ESM.pdf]

nature portfolio

Corresponding author(s): Dr. Mathieu-Benoit voisin

Last updated by author(s): Oct 11, 2022

Reporting Summary

Nature Portfolio wishes to improve the reproducibility of the work that we publish. This form provides structure for consistency and transparency in reporting. For further information on Nature Portfolio policies, see our Editorial Policies and the Editorial Policy Checklist.

Statistics

For all statistical analyses, confirm that the following items are present in the figure legend, table legend, main text, or Methods section.

n/a	Confirmed	
☐	☒	The exact sample size (*n*) for each experimental group/condition, given as a discrete number and unit of measurement
☐	☒	A statement on whether measurements were taken from distinct samples or whether the same sample was measured repeatedly
☐	☒	The statistical test(s) used AND whether they are one- or two-sided<br>*Only common tests should be described solely by name; describe more complex techniques in the Methods section.*
☒	☐	A description of all covariates tested
☐	☒	A description of any assumptions or corrections, such as tests of normality and adjustment for multiple comparisons
☐	☒	A full description of the statistical parameters including central tendency (e.g. means) or other basic estimates (e.g. regression coefficient) AND variation (e.g. standard deviation) or associated estimates of uncertainty (e.g. confidence intervals)
☐	☒	For null hypothesis testing, the test statistic (e.g. *F*, *t*, *r*) with confidence intervals, effect sizes, degrees of freedom and *P* value noted<br>*Give P values as exact values whenever suitable.*
☒	☐	For Bayesian analysis, information on the choice of priors and Markov chain Monte Carlo settings
☒	☐	For hierarchical and complex designs, identification of the appropriate level for tests and full reporting of outcomes
☒	☐	Estimates of effect sizes (e.g. Cohen's *d*, Pearson's *r*), indicating how they were calculated

Our web collection on statistics for biologists contains articles on many of the points above.

Software and code

Policy information about availability of computer code

Data collection	LEICA LAS version X<br>ZEN blue 2.6<br>DIVA v9
Data analysis	GraphPad Prism v8 & v9<br>FlowJo VX<br>Imaris v8 and v9<br>Huygens software

For manuscripts utilizing custom algorithms or software that are central to the research but not yet described in published literature, software must be made available to editors and reviewers. We strongly encourage code deposition in a community repository (e.g. GitHub). See the Nature Portfolio guidelines for submitting code & software for further information.

Data

Policy information about availability of data

All manuscripts must include a data availability statement. This statement should provide the following information, where applicable:

- Accession codes, unique identifiers, or web links for publicly available datasets
- A description of any restrictions on data availability
- For clinical datasets or third party data, please ensure that the statement adheres to our policy

Source data are provided with this paper. Raw image files are stored on servers at William Harvey Research Institute, Queen Mary University of London due to their large size. All raw Ddata of from the study are available from the corresponding author upon request.

Human research participants

Policy information about studies involving human research participants and Sex and Gender in Research.

Reporting on sex and gender	N/A
Population characteristics	N/A
Recruitment	N/A
Ethics oversight	N/A

Note that full information on the approval of the study protocol must also be provided in the manuscript.

Field-specific reporting

Please select the one below that is the best fit for your research. If you are not sure, read the appropriate sections before making your selection.

☒ Life sciences ☐ Behavioural & social sciences ☐ Ecological, evolutionary & environmental sciences

For a reference copy of the document with all sections, see nature.com/documents/nr-reporting-summary-flat.pdf

Life sciences study design

All studies must disclose on these points even when the disclosure is negative.

Sample size	Sample size is indicated in the figure legend for each experiments. In vivo experiments, we used mouse numbers that are sufficient for power calculation. The level of significance was set at 5%, and the power was set at 80%.<br>For cell-based quantitative experiments, sample sizes were based on previous similar experimental designs (Woodfin et al. Nat Immunol 2011, Proebst et al. J Exp Med 2012, Girbl et al. Immunity 2018), results of multiple independent biological replicates were used (at least 3).
Data exclusions	No data exclusion
Replication	Each in vivo experiment were performed with at least n = 4 mice per group. Presented experiments were repeated for a minimum of three times independently with reproducible results. All attempts at replication were successful
Randomization	Mice used in the present study were randomly assigned to each group.
Blinding	The researcher performing the animal experiment was blind for the animal groups. Mouse stimulation and data collection were performed by different individuals. Data was decoded after analysis.

Reporting for specific materials, systems and methods

We require information from authors about some types of materials, experimental systems and methods used in many studies. Here, indicate whether each material, system or method listed is relevant to your study. If you are not sure if a list item applies to your research, read the appropriate section before selecting a response.

Materials & experimental systems

n/a	Involved in the study	
☐	☒	Antibodies
☒	☐	Eukaryotic cell lines
☒	☐	Palaeontology and archaeology
☐	☒	Animals and other organisms
☒	☐	Clinical data
☒	☐	Dual use research of concern

Methods

n/a	Involved in the study	
☒	☐	ChIP-seq
☐	☒	Flow cytometry
☒	☐	MRI-based neuroimaging

Antibodies

Antibodies used	Anti-CD31 (390,Cat# 16-0311-85), mAbs from ThermoFisher; PB- & BV711-anti-mouse CD45 (30-F11, cat#103126100 & 10314750), AF488-anti-CD115 (AFS98, cat#135512), PE-Cy7-anti-CD31 (390, cat#102418), AF488- & AF647-anti-CD54 (YN1/1.7.4, cat#116112 &116114), APC-anti-CD140b (18A2, cat#136007), APC-Cy7-anti-CD115 (AFS98, cat#135532), AF647- & PE-Cy7-anti-CD117 (2B8, cat#105818 & 105814), PB-FceRI (MAR1, cat#), AF647-anti-IL-17A (TC11-18H10.1, cat#506912), AF594-anti-CD4 (GK1.5, cat#100446), AF700-anti-CD3 (17A2, cat#100216), BV605-anti-CD41 (MWReg30, cat#133921), PE-anti-CD49d (R1-2, cat#103608), AF647-anti-CD11c (N418, cat#117312),Rat IgG1 mAbs from Biolegend; Anti-IL-17RA (G-9, cat#sc-376374) from SantaCruz; Desmin (D33, cat#M076001-2) were obtained from Dako; Anti-aSMA (1A4, cat#A5228-200UL) from Sigma-Aldrich; anti-mouse CXCL1 (polyclonal, cat#Cat# AF-453-NA) from R&D systems. Anti-MRP14 mAb was a gift form Dr Nancy Hogg (The Francis Crick Institute, UK).
Validation	Validation of all primary commercial antibodies for the species and application was warranted by the vendors. Validation statement can be found on the manufacturers' website. In vivo labeling of endothelial cells with anti-CD31 mAb have been validated previously (Woodfin et al. Nat Immnuol 2011). MC labeling using anti-CD117 mAb was confirmed using MC reporter mice (i.e. Mcpt5-Cre-YFP) as discussed in the study. Anti-MRP14 was validated in house with co-localisation with reporter neutrophil mice and other antibodies (i.e. anti-Ly6G).

Animals and other research organisms

Policy information about studies involving animals; ARRIVE guidelines recommended for reporting animal research, and Sex and Gender in Research

Laboratory animals	Male WT C57BL/6 (stock number JAX #000664, Charles River, UK), LysM-EGFP-ki (Gift from Dr M. Sperandio (Ludwig Maximilians University Munich, Germany 59), α-SMA-RFPcherry-Tg (Gift from Dr D. Rowe (University of Connecticut Health Center, US), LysM-EGFP-ki; α-SMA-RFPcherry-Tg mice (8-12 weeks old) were used for all studies. Mcpt5-Cre-ROSA26-YFP and Mcpt5-Cre-RDTA/RDTA were provided by A. Roers (Institute for Immunology, Heidelberg University Hospital, Heidelberg, Germany) and generated as previously described. Mcpt5-Cre-RDTA/RDTA were crossed with LysM-EGFP-ki; α-SMA-RFPcherry-Tg animals to generate Mcpt5-Cre-RDTA;LysM-EGFP-ki; α-SMA-RFPcherry-Tg. Mcpt5-Cre-RDTA;LysM-EGFP-ki; α-SMA-RFPcherry-Tg expressing the Cre recombinase were referred as MCdeficient, littermates Mcpt5-Cre--RDTA;LysM-EGFP-ki; α-SMA-RFPcherry-Tg (MCctrl) were used as controls. Of note, no difference in level of MC deficiency was observed between Mcpt5-Cre+-RDTA/RDTA (0.0±0.06) and Mcpt5-Cre+-RDTA/- mice (0.0±0.00). Il17atm1.1(icre)Stck (stock number JAX strain #016879) mice, referred as “IL-17AKO”, were purchased from Jackson Laboratory (Maine, US) and generated as previously described 60. In these animals the endogenous Il17a gene has been substituted with a Cre-recombinase gene insert inducing total IL-17A deficiency in homozygous mice.<br>MC reconstitution was performed as previously described 25. Briefly, bone-marrow derived MCs (BMMCs) were derived from WT or Il17atm1.1(icre)Stck mice. Bone marrow cells were isolated from the femur of animals. After 3 weeks of differentiation in presence of 10 ng/ml of interleukin 3, mature BMMCs, validated by flow cytometry for high expression of Fcε⍰RI and CD117, were injected i.v. (106) and locally in the scrotal (i.s.) cavity (106). Four months post-engraftment, mice subjected to TNF-stimulation were analysed for neutrophil infiltration, ICAM-1 and CXCL1 expression. No tissue-infiltrated neutrophils were observed in mice injected with BMMCs at steady state. BMMCs from WT or Il17atm1.1(icre)Stck mice showed similar level of purity (~98%) and expression level of Fcε⍰RI and CD117. All animals were group housed in individually ventilated cages (maximum of 5 mice per cage) under specific pathogen-free (SPF) conditions and a 12-hour (h) light-dark cycle. Room temperature and humidity were maintained within 18-20°C and 30-70% humidity. Food and water were provided ad libitum. At the end of the experiments, mice were euthanised using cervical dislocation.<br>All in vivo experiments were conducted at the William Harvey Research Institute, Queen Mary University of London, UK under the UK legislation for animal experimentation (UK Home Office licence number PPL: P873F4263) and in agreement with the UK Home Office Animals Scientific Procedures Act 1986 (ASPA).
Wild animals	No wild animals were used in the study
Reporting on sex	Most of the study was performed using both male and females mice. However the intravital experiments were performed in the cremaster muscle of the mouse testes.
Field-collected samples	No field collected samples were used in the study
Ethics oversight	All in vivo experiments were conducted under the UK legislation according to the Animal Scientific Procedures Act 1986, with all procedures being conducted in accordance with UK Home Office regulations.

Note that full information on the approval of the study protocol must also be provided in the manuscript.

Flow Cytometry

Plots

Confirm that:

☒ The axis labels state the marker and fluorochrome used (e.g. CD4-FITC).

☒ The axis scales are clearly visible. Include numbers along axes only for bottom left plot of group (a 'group' is an analysis of identical markers).

☒ All plots are contour plots with outliers or pseudocolor plots.

☒ A numerical value for number of cells or percentage (with statistics) is provided.

Methodology

Sample preparation	Whole blood was collected through the hepatic vein in PBS + 50 mM EDTA. Indicated organs were harvested, mechanically dissociated and treated with 625 U/mL Collagenase I (ThermoFisher) and 100 U/ml DNAse I (Sigma-Aldrich) for 30min at 37° C. Where required, samples were treated with ACK buffer (150 mM NH3Cl, 1 mM KHCO3 and 1 mM EDTA) to lyse red blood cells. Subsequently, single cell suspensions were incubated with anti-CD16/-CD32 antibodies (Becton Dickinson) to block Fc-receptors and stained with primary fluorescently labelled antibodies of interest. Dead cells were excluded using Zombie Aqua (Biolegend)
Instrument	LSR Fortessa flow cytometer (Becton Dickinson)
Software	FlowJo software (TreeStar)
Cell population abundance	Mast cells, endothelial cells, pericytes abondance were dependant of the organ analysed. In vitro pericyte purity was ~95% post sorting.
Gating strategy	Gating strategy are indicated in supplementary S3, S5 and S6. All gating strategy followed the same pattern: singlets (FSC-A/FSC-H), debris exclusion, removal of dead cells (negativity for viability marker)

☒ Tick this box to confirm that a figure exemplifying the gating strategy is provided in the Supplementary Information.
